# Supplementary material for: Stress-induced red nucleus attenuation induces anxiety-like behavior and lymph node CCL5 secretion
Source: Nat Commun. 2023 Oct 30;14:6923. doi: 10.1038/s41467-023-42814-1 (PMC10616295; doi:10.1038/s41467-023-42814-1)
Supplement: Supplementary file 1 — Supplementary Information [file 41467_2023_42814_MOESM1_ESM.pdf]

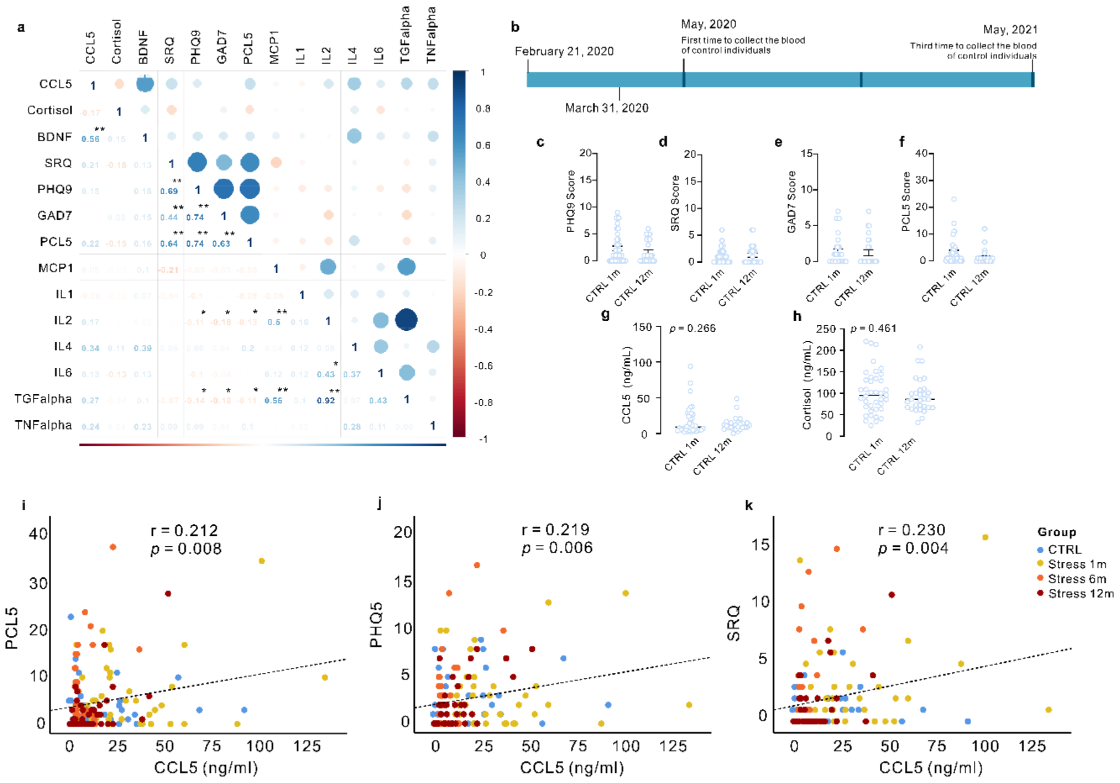

Fig. S1 Associations of immune mediators and cortisol with mental health outcomes. a, Spearman's correlation coefficient was calculated. Red bubbles indicate a negative relationship, and blue bubbles indicate a positive relationship. The bubble size indicates the p value, and a larger bubble size indicates a smaller p value. The data were Bonferroni corrected. **b,** Flowchart of blood collection from controls. **c-f,** One-year follow-up study found that the PHQ-9, GAD-7, SRQ-20, and PCL-5 scores in the control group were in line with baseline concentrations. **g&h,** One-year follow-up study found that CCL5 and cortisol concentrations in control individuals were in line with baseline concentrations. **i-k,** Correlation between CCL5 in plasma and PCL-5, PHQ-5 and SRQ-20 scores in controls and stressed individuals. The data were Bonferroni corrected. Data are expressed as the mean ± SEM. * $p < 0.05$, ** $p < 0.01$, *** $p < 0.001$ vs. ctrl.

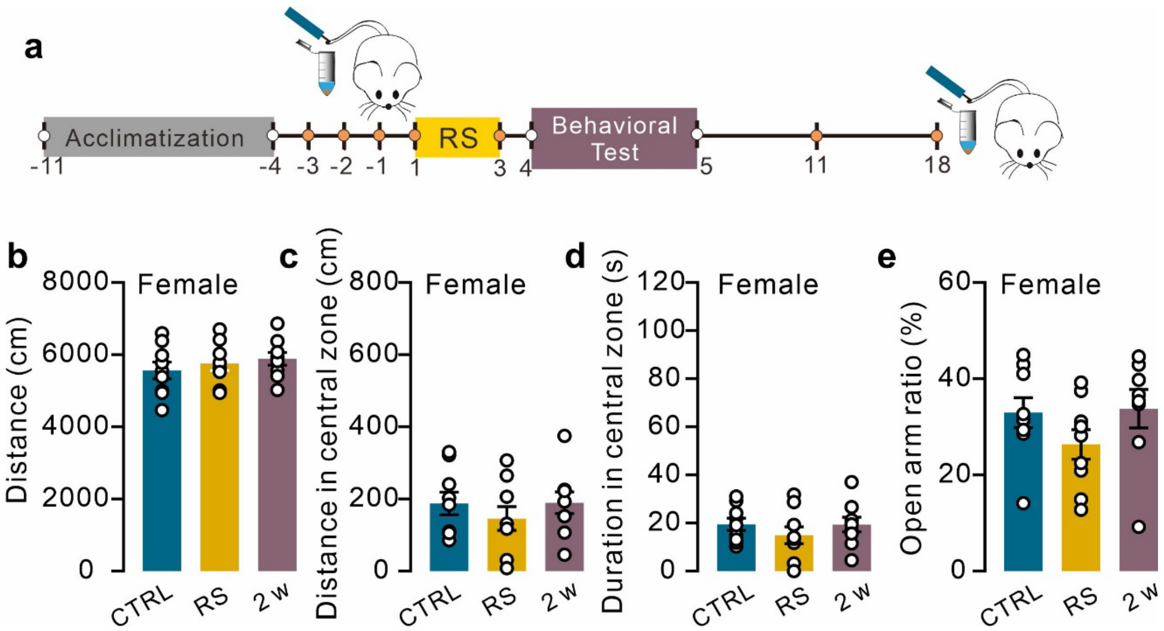

Fig. S2 Acute restraint stress caused a dramatic increase in CCL5 but did not trigger anxiety-like behaviors in female rats. a, Schematic timeline and behavioral paradigm in female rats, RS: restraint stress. **b,** No difference between control rats and RS rats in distance moved in the open field test. **c,** The distance moved in the central zone. **d,** The time spent in the central zone in the open field test was not different between RS rats and control rats. **e,** There was no difference in the open arm ratio in the elevated plus maze test. * $p < 0.05$, ** $p < 0.01$, *** $p < 0.001$ vs. ctrl.

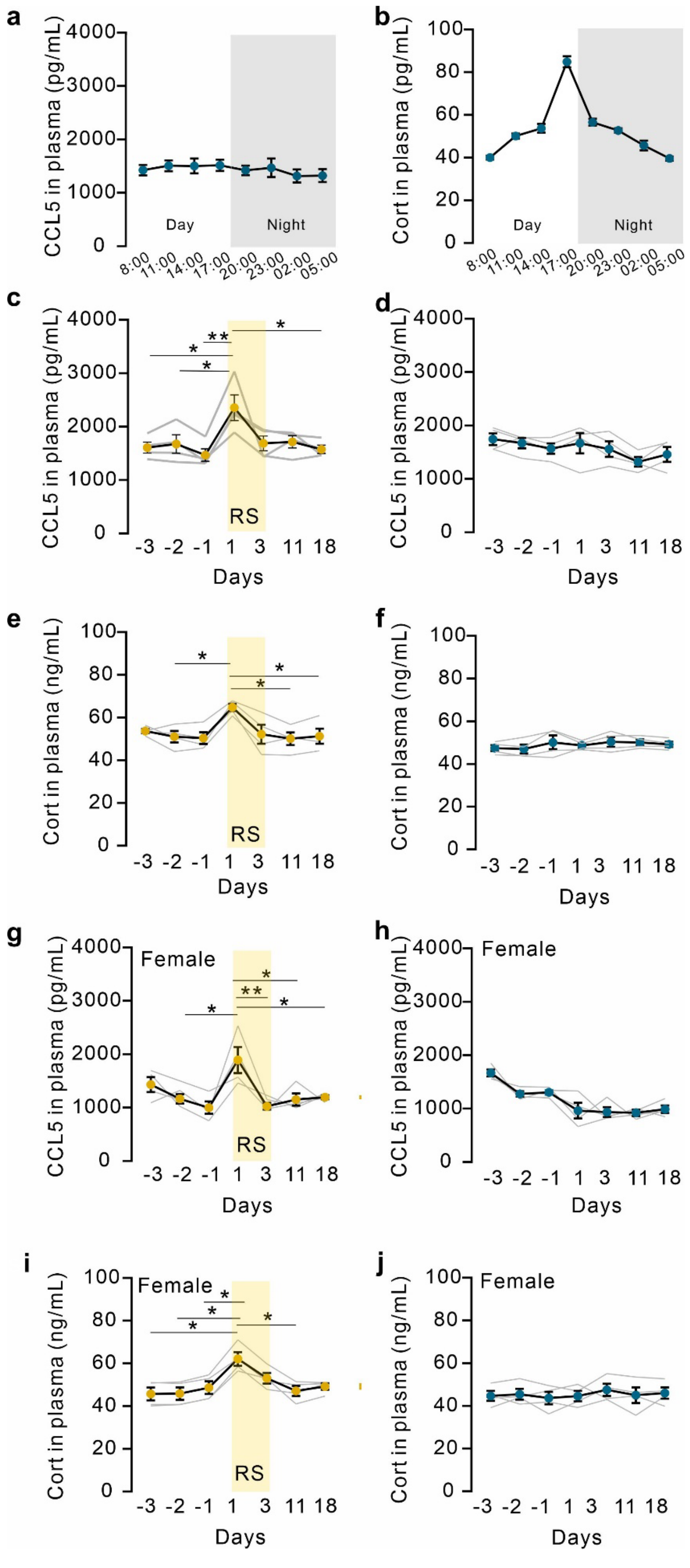

Fig. S3 CCL5 and corticosterone level fluctuation in adult rats. a, Twenty-four-hour CCL5 profile: CCL5 levels in serum were not affected by circadian rhythms. **b,**

Twenty-four-hour corticosterone profile: cortisol levels in serum affected by circadian rhythms. **c,** CCL5 level fluctuation before, during and after restraint stress exposure in rats. **d,** CCL5 levels in control rats. **e,** Corticosterone level fluctuation before, during and after restraint stress exposure in rats. **f,** Corticosterone levels in control rats. **g,** CCL5 level fluctuation before, during and after restraint stress exposure in female rats. **h,** CCL5 levels in control female rats. **i,** Corticosterone level fluctuation before, during and after restraint stress exposure in female rats. **j,** Corticosterone levels in control female rats. Data are expressed as the mean ± SEM. Data are expressed as the mean ± SEM. * $p < 0.05$, ** $p < 0.01$, *** $p < 0.001$ vs. ctrl.

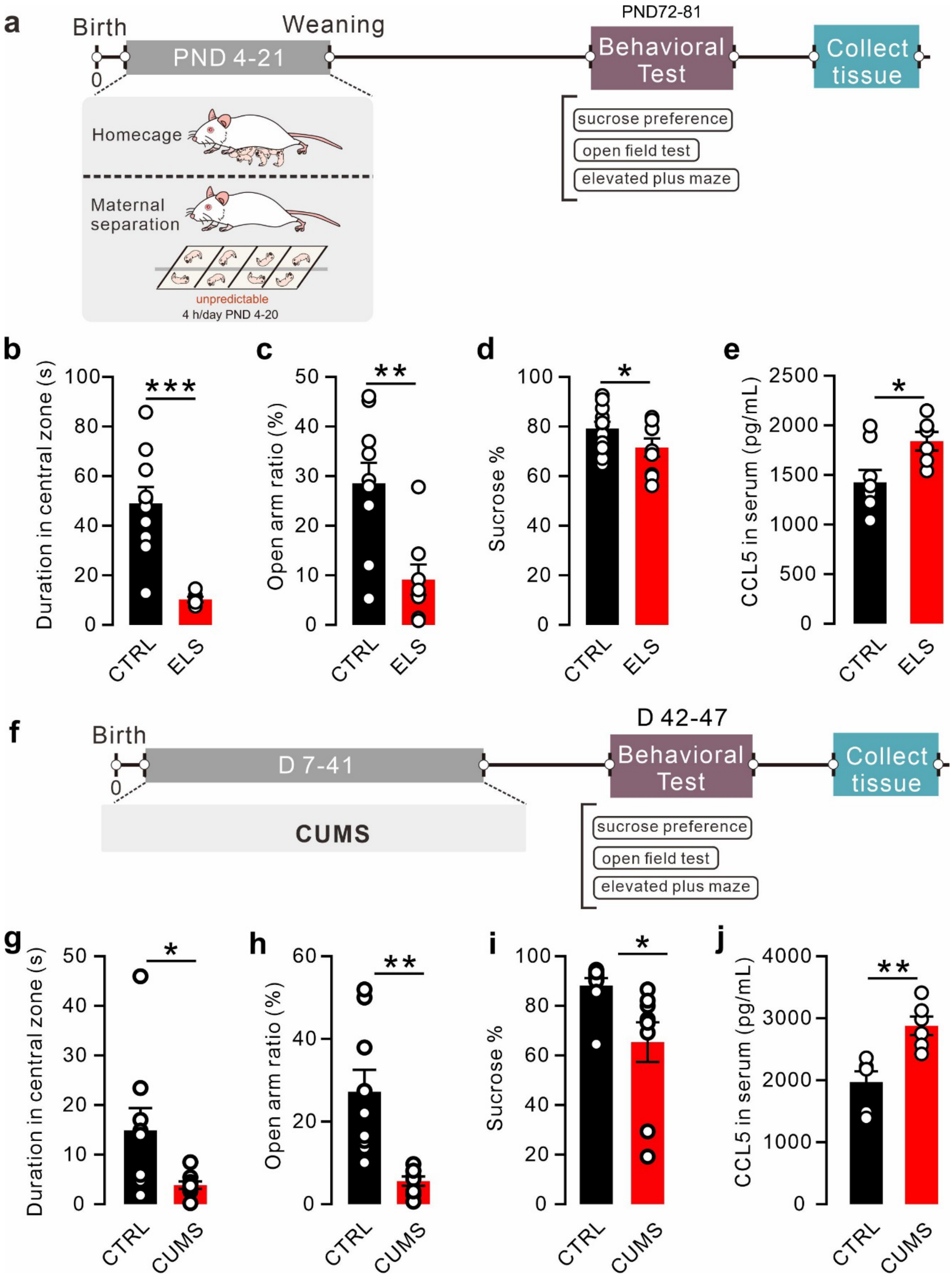

Fig. S4 Both early life stress (ELS) and chronic mild unpredictable stress (CUMS) led to anxiety-like behavior and a sharp increase in CCL5 in rats. a, Schematic timeline of the ELS and behavioral paradigm in rats, PND: postnatal days. **b-c,** Rats that experienced ELS spent less time in the central zone in the OFT and open arms in the EPM (two-tailed unpaired test). **d,** ELS-treated rats showed less

sucrose preference than controls (two-tailed unpaired test). **e,** CCL5 levels in the serum of ELS-treated rats were significantly higher than those of controls (two-tailed unpaired test). **f,** Schematic timeline of the CUMS and behavioral paradigm in rats, CUMS: chronic unpredictable mild stress. **g-h,** Rats that experienced CUMS spent less time in the central zone in the OFT and open arms in the EPM (two-tailed unpaired test). **i,** CUMS-treated rats showed less sucrose preference than controls (two-tailed unpaired test). **j,** CCL5 levels in the serum of CUMS-treated rats were significantly higher than those of controls (two-tailed unpaired test). Data are presented as the mean ± SEM. *$P < 0.05$, **$P < 0.01$, ***$P < 0.001$.

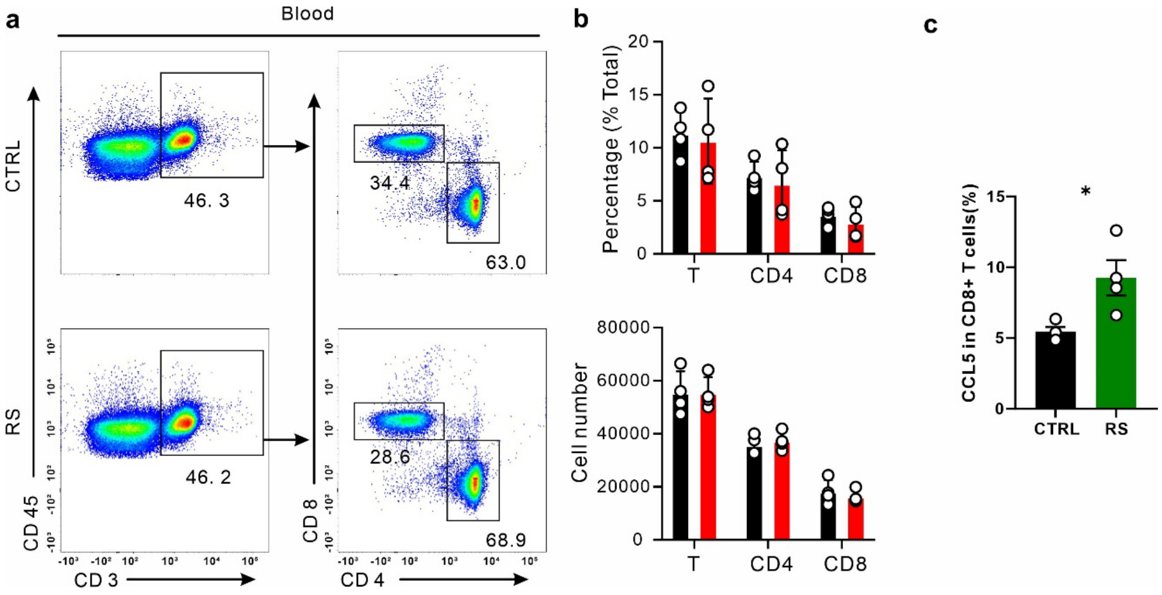

Fig. S5 Flow cytometry analysis of the frequency and absolute numbers of different T-cell populations in the blood of control and restraint stress-treated rats (n = 5 per group). **c,** CD8+ T cells were isolated from the CLNs of CTRL and RS rats, and incubated with monensin (1ug/mL) for 4 h before harvest. Flow cytometric analysis of the percentage of CCL5-producing CD8+ T cells (n = 4).

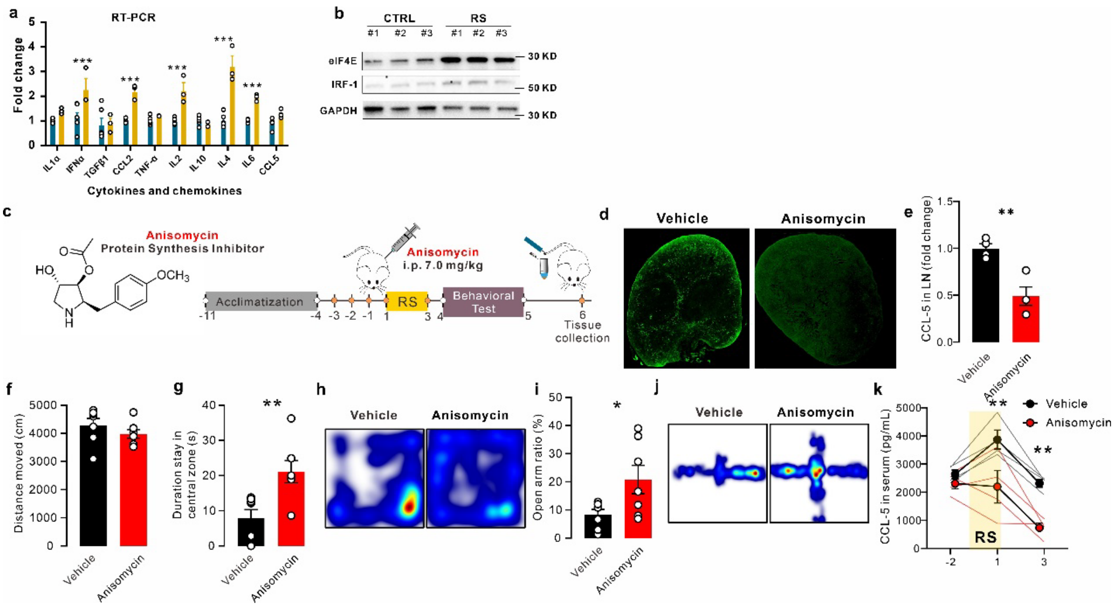

Fig. S6 Stress-induced translation activation promoted CCL5 protein synthesis in lymph nodes. a, RT–PCR revealed that restraint stress increased the levels of cytokines and chemokines in the adult rat cervical lymph nodes (two-way ANOVA, interaction $F_{9,73}$ = 9.856, P < 0.0001, effect of cytokines/chemokines $F_{9,73}$ = 12.20, P < 0.0001, effect of restraint stress $F_{1,73}$ = 117.8, P < 0.0001; n = 4 per group). **b,** Western blot (WB) analyses of the indicated proteins in cervical lymph nodes. #1, repeat 1; #2, repeat 2; #3, repeat 3. **c,** Schematic timeline and behavioral paradigm for restraint stress-treated rats. **d,** The expression of CCL5 in cervical lymph nodes was visualized using immunofluorescence staining. Representative confocal images are shown. Bar, 500 μm. **e,** The expression of CCL5 in cervical lymph nodes was analyzed using RT-PCT (two-tailed unpaired t test, t = 4.578, df = 6, P = 0.0038; n = 4 per group). **f,** There was no difference in locomotion ability between vehicle (i.p. saline) and anisomycin rats (two-tailed unpaired t test, t = 1.096, df = 12, P = 0.2948; n = 7 per group). **g-h,** The time spent in the central zone in rats treated with anisomycin was increased significantly compared to that in rats treated with vehicle (two-tailed unpaired t test, t = 3.337, df = 12, P = 0.0059; n = 7 per group). **i-j,** rats treated with anisomycin spent more time in the open arms in the EPM than rats treated with saline (two-tailed unpaired t test, t = 2.313, df = 12, P = 0.0393; n = 7 per group). **k,** Restraint stress led to a sharp increase in CCL5 in serum in vehicle rats but not in anisomycin rats (two-way ANOVA, interaction $F_{2,18}$ = 3.069, P = 0.0713, effect of restraint stress $F_{2,18}$ = 12.55, P = 0.0004, effect of anisomycin $F_{1,18}$ = 23.00,

$P = 0.0001$; n = 4 per group). Data are presented as the mean ± SEM. *$P < 0.05$, **$P < 0.01$, ***$P < 0.001$.

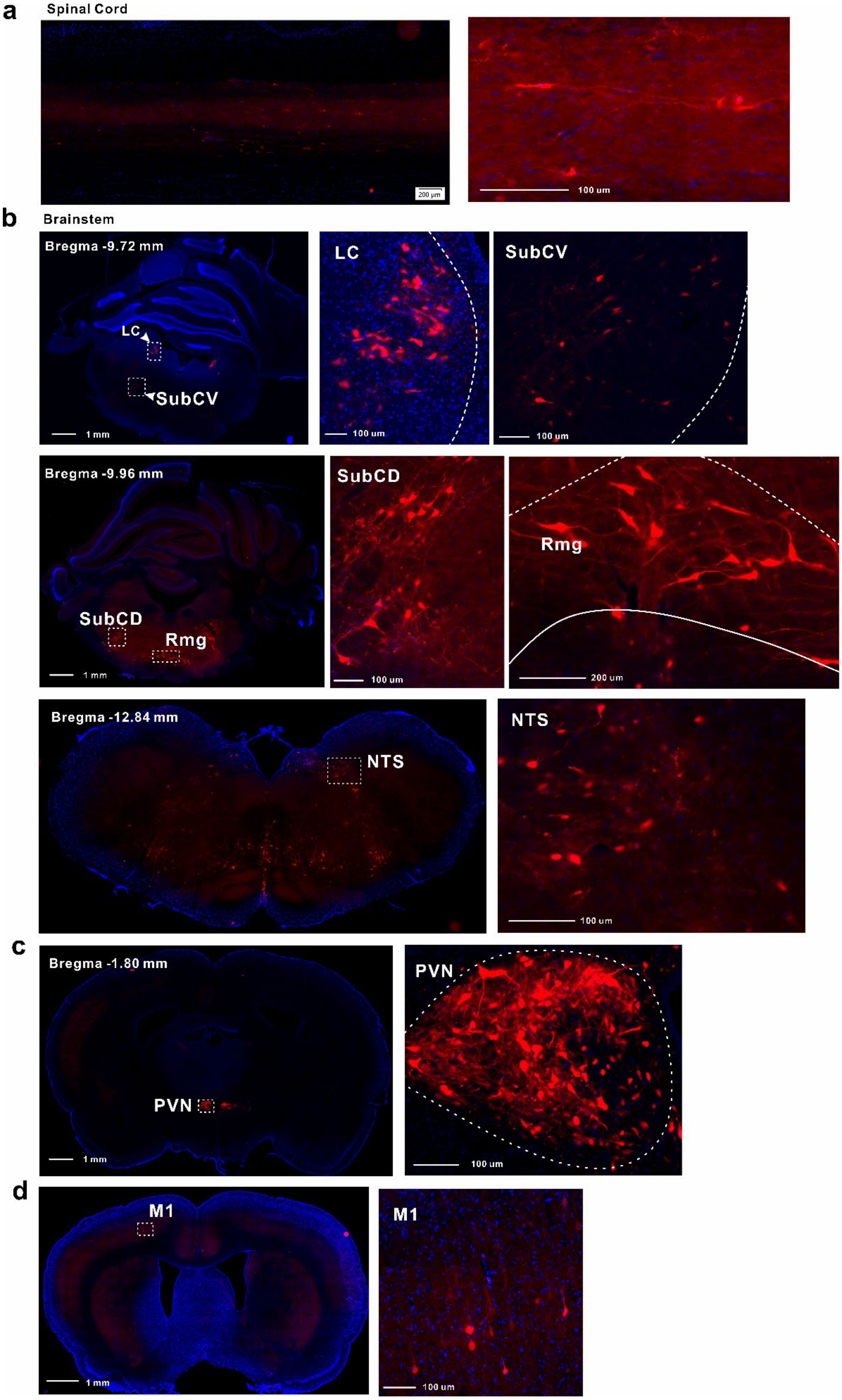

Fig. S7 Retrograde PRV tracing from CLNs. a-d, Selected CNS regions that were labeled by PRV. LC, locus coeruleus; SubCV, subcoeruleus nucleus, ventral part; SubCD, subcoeruleus nucleus, dorsal part; RMg, raphe magnus nucleus; NTS, nucleus tractus solitarius; PVN, paraventricular thalamic nucleus; M1, primary motor cortex.

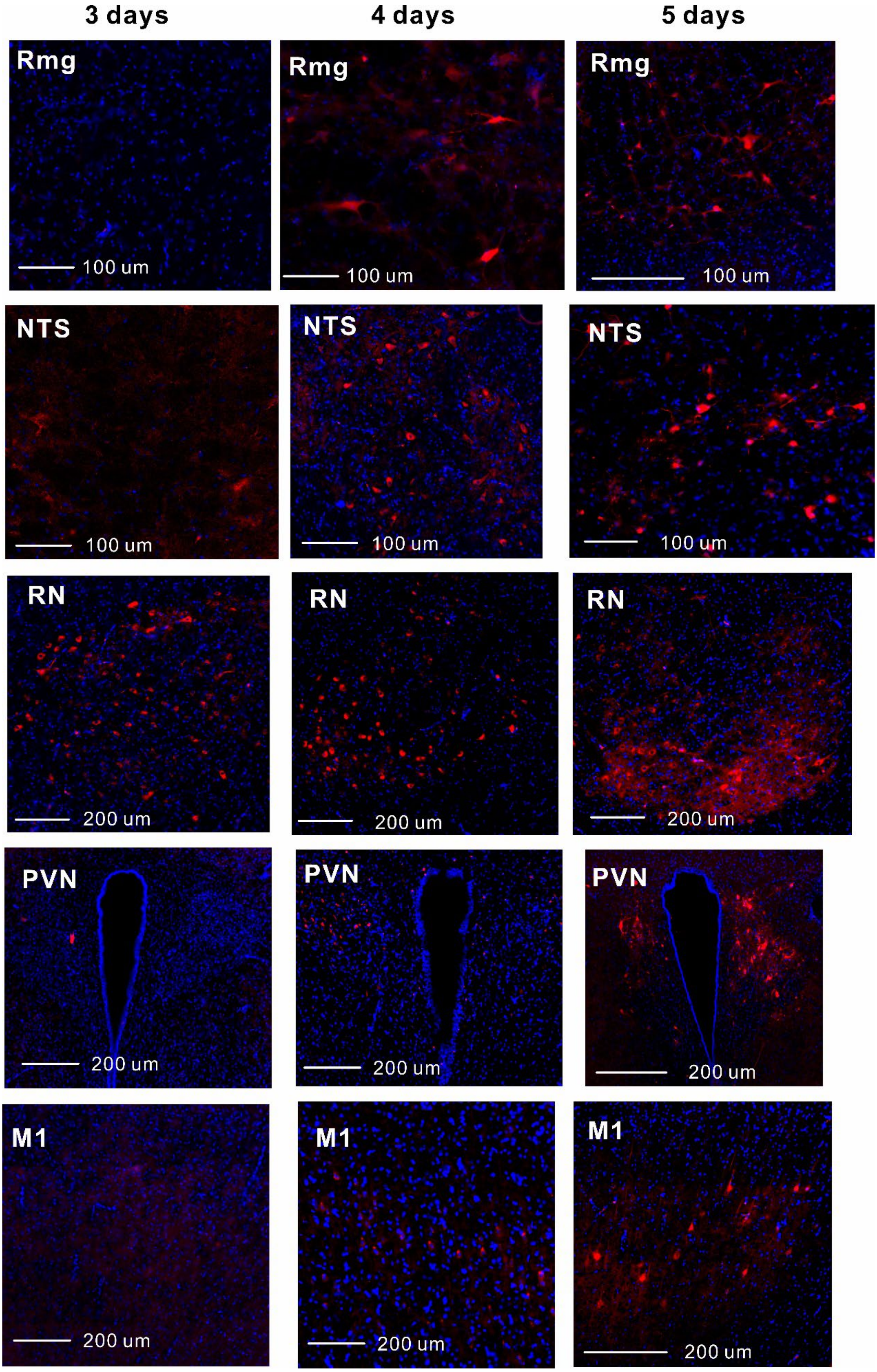

Fig. S8 Time-dependent infection of different brain areas after PRV injection into CLNs. Representative images showing PRV-infected neurons (red) in different

brain areas at different time points after viral injection. Left: 3 days; middle 4 days; right: 5 days. These experiments were performed in 3 male rats for each group.

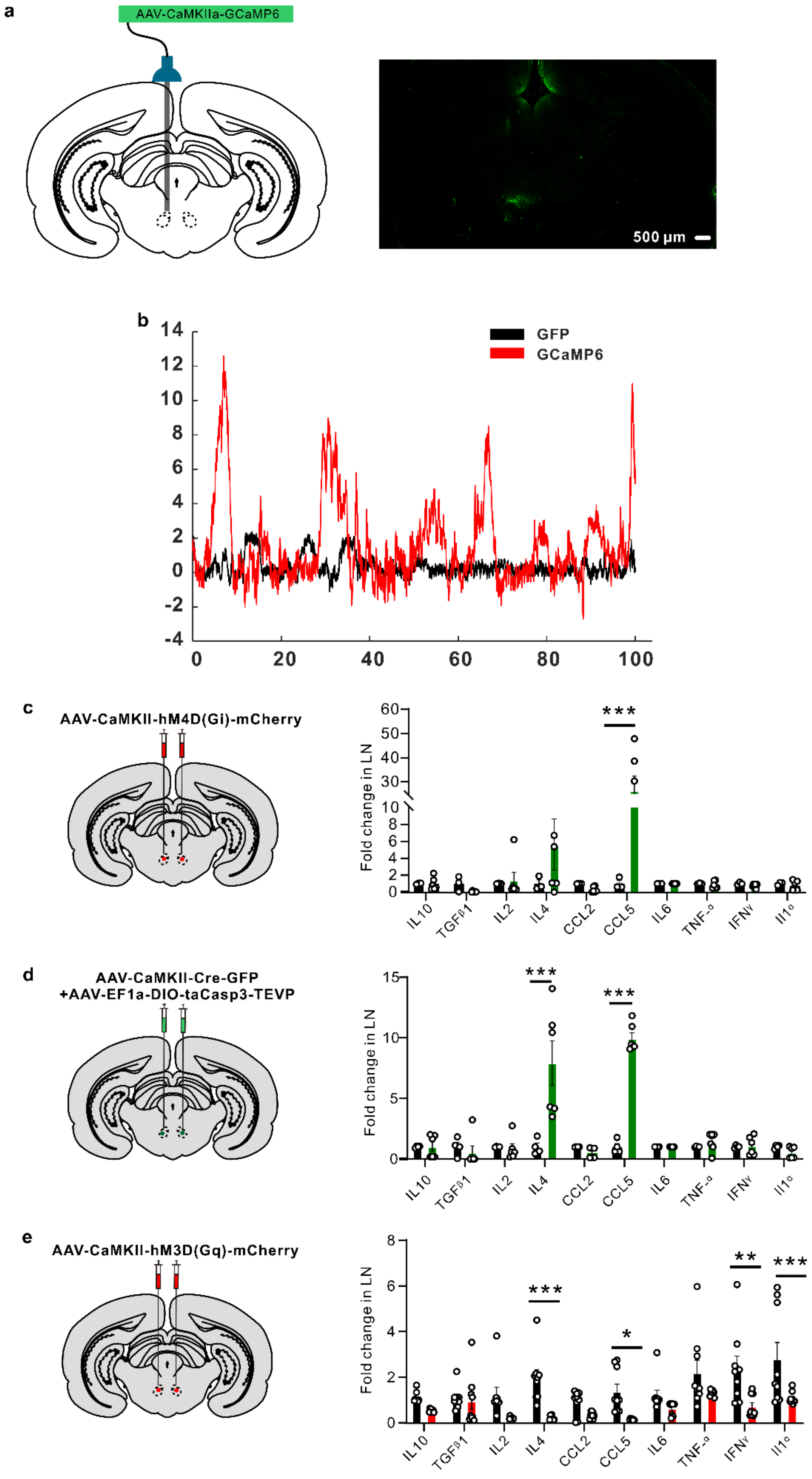

Fig. S9 Optical recording of RN glutamatergic neuron activity in awake freely behaving rats. a, Expression of GCaMP6 in the RN of adult rats. **b,** fluorescence

traces during open field exploration from RN^{GCaMP6} rats (red) and RN^{GFP} rats (black). **c,** Inflammatory gene levels in control and hM4Di rats after CNO injection (two-way ANOVA, P < 0.0001). **d,** Inflammatory gene levels in control and taCasp3 rats (two-way ANOVA, left to right: *P* < 0.0001, < 0.0001). **e,** Inflammatory gene levels in control and hM3Dq stress-treated rats after CNO injection (two-way ANOVA, left to right: P < 0.0001, < 0.0001, 0.0215, < 0.0001). Data are presented as the mean ± SEM. *$P < 0.05$, **$P < 0.01$, ***$P < 0.001$.

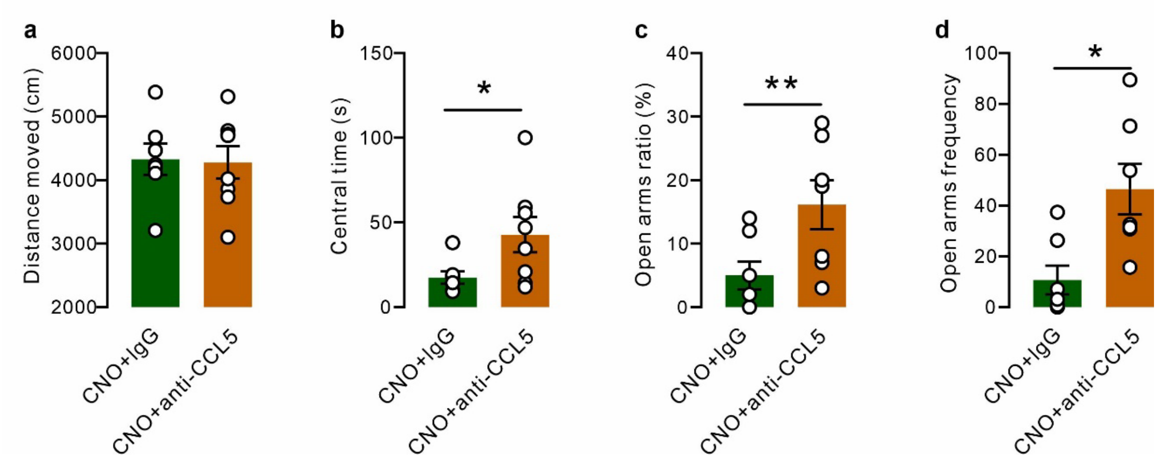

Fig. S10 CCL5 neutralization reversed anxiety induced by RN inhibition. a, Distance moved in OFT (two-tailed unpaired t test, t = 0.1369, *P* = 0.8932, n = 8 rats per group). b, The time spent in the central zone in the OFT was significantly increased in anti-CCL5 rats compared to controls (two-tailed unpaired t test, t = 2.176, *P* = 0.0486, n = 8 rats per). c-d, Both the open arm ratio and frequency were increased in anti-CCL5 rats compared to IgG rats (two-tailed unpaired t test, t = 3.129; 2.511, *P* = 0.0087; 0.0273, n = 8; 8 rats per). Data are presented as the mean ± SEM. *P < 0.05, **P < 0.01, ***P< 0.001.

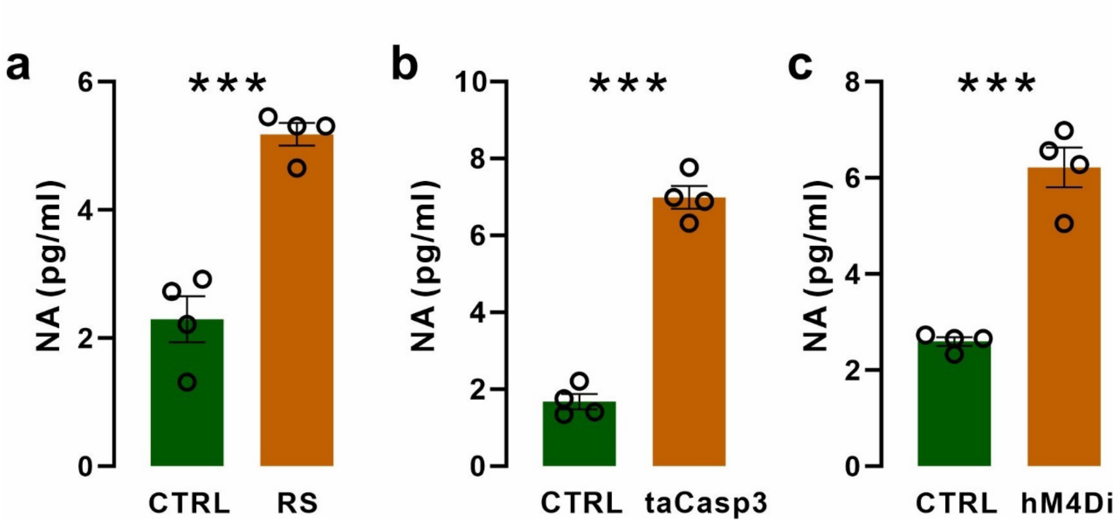

Fig. S11 NA was significantly increased in CLNs both in stressed rats and in RN inhibited rats. a. NA was increased significantly in RS rats compared to controls (two-tailed unpaired t test, t = 7.201, *P* = 0.0004, n = 4 rats per group). b, Ablating RN glutamatergic neurons increased the NA level in CLNs (two-tailed unpaired t test, t = 14.83, P < 0.0001, n = 4 rats. c. Inhibiting RN glutamatergic neurons led to a upward trend in NA level in CLNs (two-tailed unpaired t test, t = 8.511, *P* = 0.0001, n = 4 rats per group).). Data are presented as the mean ± SEM. *P < 0.05, **P < 0.01, ***P< 0.001.

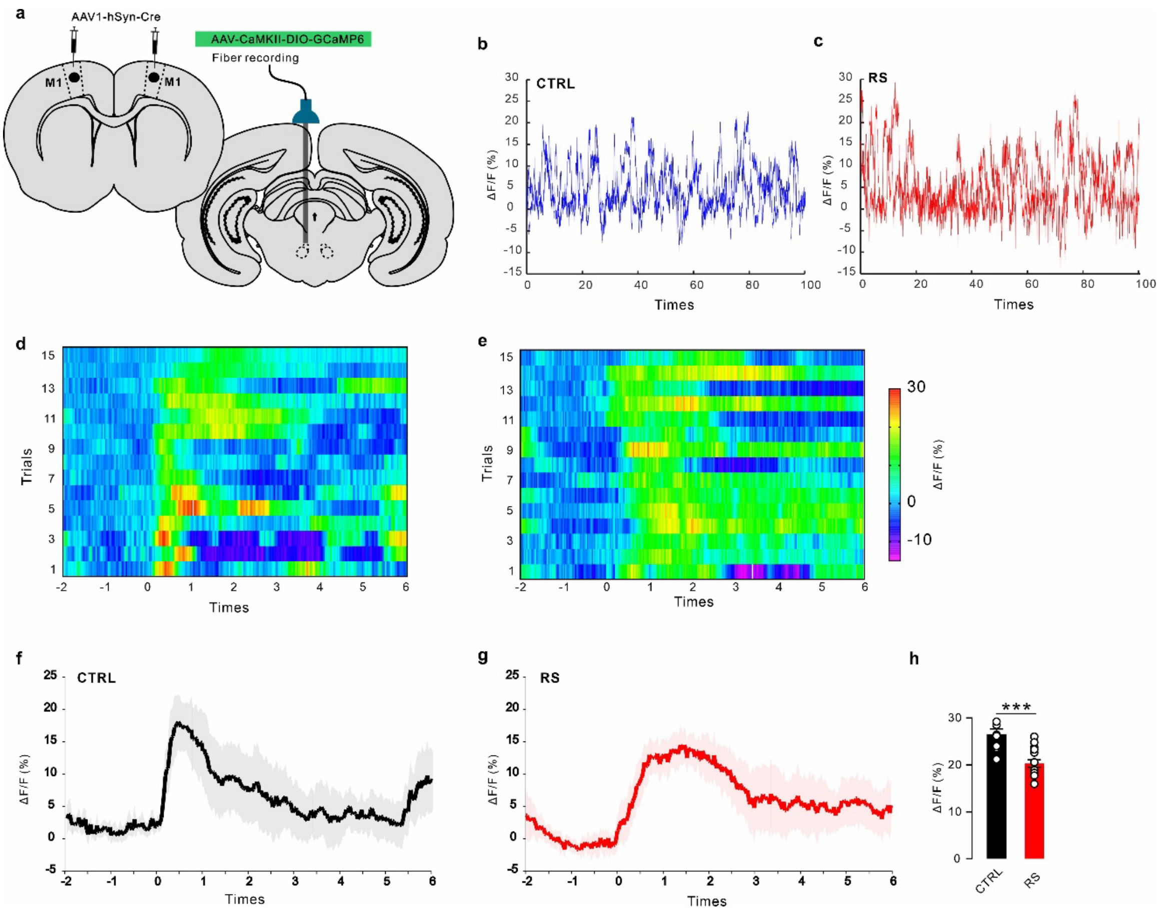

Fig. S12 Optical recording of M1-RN glutamatergic neuron activity in awake freely behaving rats. a, Injection of AAV1-hSyn-Cre into the M1 and AAV-CaMKII-DIO-GCaMP6 into the RN. **b-c,** GCaMP6 signals from M1-RN glutamatergic neurons aligned to the moment of exploratory behavior to the central zone in the EPM. Control: gray; RS: red. **d-e,** Heatmap showing the Ca^{2+} signals evoked by exploratory behavior in the EPM in M1-RN glutamatergic neurons from control and RS-treated rats. **f-g,** Averaged responses of control and RS-treated rats (black and red line, mean calcium signal during exploratory behavior in EPM; gray area and red area, SEM). **h,** Quantification of the change in calcium signals from exploratory behavior in control and RS-treated rats (two-tailed unpaired t test, n = 6 rats per group).

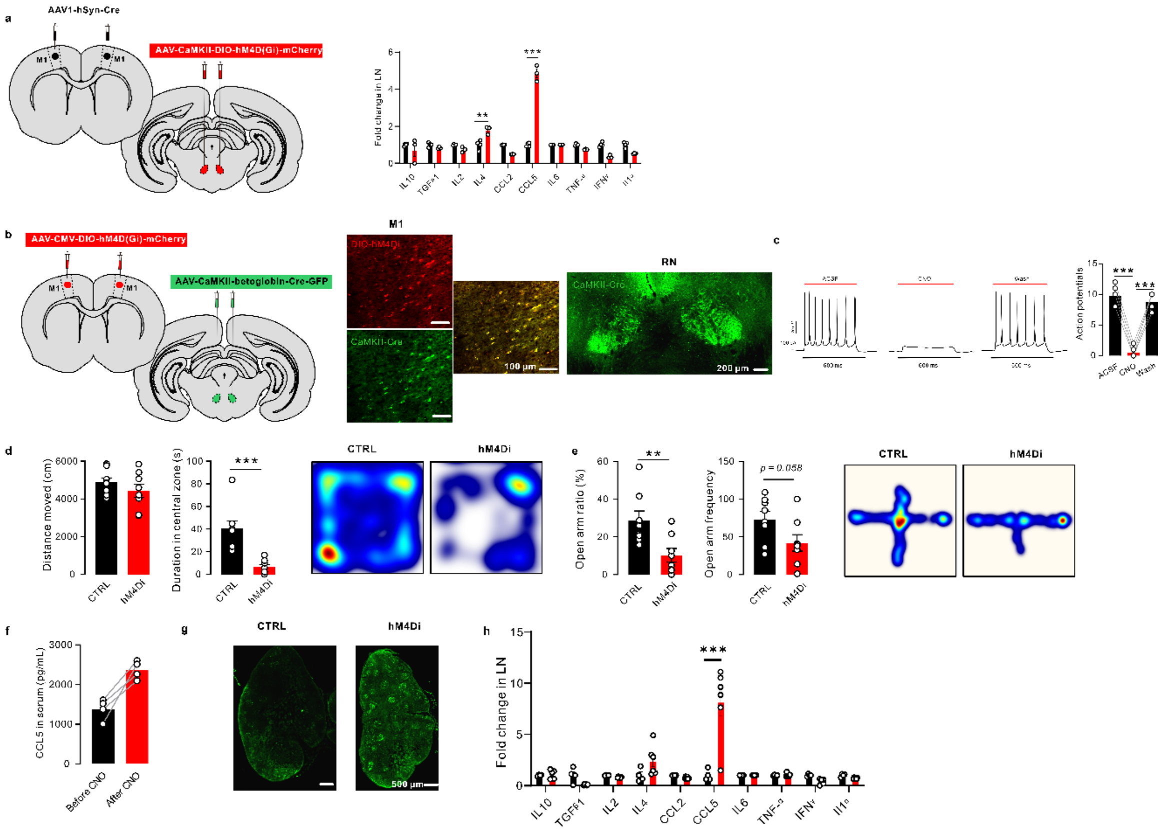

Fig. S13 RN glutamatergic neurons triggered anxiety-like behavior after receiving signals through M1 projections. a, Inflammatory gene levels in control and hM4Di rats after CNO injection (two-way ANOVA, left to right: $P = 0.0396$, 0.0153, < 0.0001, 0.0485, < 0.0001, < 0.0001). **b,** Left: injection of AAV-DIO-hM4Di-mCherry into the M1 and AAV-CaMKII-betaglobin-cre-GFP into the RN. Right: a representative confocal image of hM4Di^{+} neurons and CaMKII-cre-GFP^{+} neurons in the M1, CaMKII-cre-GFP^{+} neurons in the RN. **c,** Current-evoked action potentials in representative hM4Di-infected neurons in the M1 recorded before, during, and after CNO perfusion (10 mM) (n = 6 neurons; two-tailed paired t test, t = 22.14, $P < 0.0001$; t = 19.76, $P < 0.0001$, respectively). **d-e,** Inhibition of M1-RN projections decreased the time spent in the central zone in the OFT and the open arm ratio in the EPM (two-tailed unpaired t test, t = 4.580, $P = 0.0004$; t = 3.030, $P = 0.0090$, n = 8 rats per group). **f-g,** CCL5 levels in the serum and CLNs were significantly increased after CNO injection. **h,** Inflammatory gene levels in control and hM4Di rats after CNO injection (two-way ANOVA, $P < 0.0001$).

a

Comparison of rsFC in stressed individuals and controls

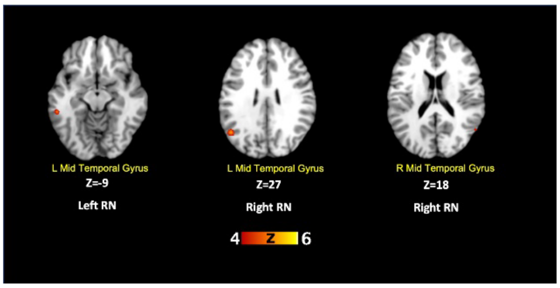

b

Resting-state Functional connectivity group comparisons

	Seeds	Brain region	BA	Voxels	MNI (x, y, z)	t	p
**Stress < Control**	**RN_L**	L Mid Temporal Gyrus	20	87	-54. -36. -9	5.10	0.001*
		L Mid Temporal Gyrus	38	35	-42, 0, -48	4.16	0.049
		L Mid Frontal Gyrus	9	35	-30, 33, 33	4.88	0.049
	**RN_R**	L Mid Tempor Gyrus	39	67	-48, -60, 27	5.27	0.002*
		R Mid Tempor Gyrus	19	60	54, -60, 18	4.40	0.002*

Note: Cluster-level FDR, $p < 0.05$. means the significance still survived after multiple comparison corrections ($p = 0.05/2$)

Fig. S14 Weakened functional connectivity of the RN in stressed individuals in the first month post stress. a, Representative images of comparison of rsFC in stressed individuals and controls. b, Resting-state functional connectivity group comparisons.

Table S1. Depression, Anxiety and Distress Measurements in Total Cohort and Subgroups

Variables	Total	Working position		P value
		Frontline in Wuhan	Second-line outside Wuhan	
PHQ-9, depression symptoms				
Mean	2.73 ± 0.345	3.28 ± 0.524	2.10 ± 0.420	.087
Median	2.00	2.00	1.00	
GAD-7, anxiety				
Mean	1.477 ± 0.220	1.66 ± 0.305	1.27 ± 0.320	.379
Median	1.00	1.00	0.00	
SRQ-20, self-reporting questionnaire				
Mean	2.01 ± 0.307	2.92 ± 0.512	0.976 ± 0.208	.001
Median	1.00	2.00	1.00	
PCL-5, post traumatic stress disorder				
Mean	4.97 ± 0.701	6.49 ± 1.11	3.22 ± 0.735	.019
Median	2.00	4.00	1.00	

Table S2. Common differentially expressed genes.

Subset of genes differentially expressed by restraint stress.

Diff Group	Diff Number
CTRL-vs-RS	2077 up
	2620 down

Table S3. Antibodies used for immunohistochemistry.

Use, target, catalogue identifier, company, concentration, and species for all antibodies.

Antibody use	Antibody target	Catalogue #	Company	Concentration	Species
IHC/primary	CCL5	710001	Thermo Fisher	1:500	mouse
WB/primary	eIF4E	66655	Proteintech	1:2000	mouse
WB/primary	IRF-1	11335	Proteintech	1:2000	rabbit
IHC/primary	vGlu1	48-2400	Invitrogen	1:300	rabbit
IHC/primary	GAD	PA5-21397	Invitrogen	1:300	rabbit
IHC/secondary	Alexa fluor 647-anti-mouse	A-21245	Invitrogen	1:5000	goat
IHC/secondary	Alexa fluor 488-anti-rabbit	1796375	Invitrogen	1:5000	donkey
IHC/secondary	Alexa fluor 555-anti-rabbit		Invitrogen	1:5000	donkey

Table S4. Primers used for qPCR and RNA-scope.

Gene name, accession number, forward primer sequence, and reverse primer sequence of primer pairs used in real-time PCR amplification of mRNA.

Rat qPCR Primers

**Gene**	**Forward-sequence**	**Reverse-sequence**
GAPDH	AAGGTCGGTGTGAACGGATT	TGAACTTGCCGTGGGTAGAG
eIF4E	CAGTGATGACGTGTGTGGAG	ATGTGTGTGACTGCATCTCTG
IRF-1	GCTCTACTCTGCCTGATGACC	AGAGACTGCTGCTGACGAC
CCL5	AGATCTCCACAGCTGCATCC	AGAGGTAGGCAAAGCAGCAG
IL6	AGAGTCACAGAAGGAGTGGC	GGTTTGCCGAGTAGACCTCAT
IL4	CCTTGCTGTCACCCTGTTCT	CGTTCTCCGTGGTGTTCCTT
IL10	CAGCAAAGGCCATTCCATCC	GCTTGGCAACCCAAGTAACC
IL2	TCTGCAGCGTGTGTTGGATT	GGCTCATCATCGAATTGGCAC
TNF-α	ATCCGAGATGTGGAACTGGC	CGATCACCCCGAAGTTCAGT
IL1α	GACAAGCCTGTGTTGCTGAAG	AAGCTGCGGATGTGAAGTAGT
CCL2	GCCTGTTGTTCACAGTTGCT	AGTTCTCCAGCCGACTCATT
TGFβ1	CCAAACTAAGGCTCGCCAGT	TAGATTGCGTTGTTGCGGTC
IFNγ	GGCAAAAGGACGGTAACACG	TTCACCTCGAACTTGGCGAT

Table S5. Sequencing quality control measures.

Sample name, total sequenced reads, and mapping rate for uniquely mapped reads for each RNA-seq sample.

RNA-seq

Sample	Total Reads	Mapping rate (%)
Ctrl-1	33846732	89.90
Ctrl-2	42099052	91.20
Ctrl-3	37539230	89.39
Ctrl-4	45131314	92.01
RS 1	45069404	92.59
RS 2	41684368	92.50
RS 3	43660900	91.92
RS 4	44390356	92.42
