# Supplementary material for: Stress-induced red nucleus attenuation induces anxiety-like behavior and lymph node CCL5 secretion
Source: Nat Commun. 2023 Oct 30;14:6923. doi: 10.1038/s41467-023-42814-1 (PMC10616295; doi:10.1038/s41467-023-42814-1)
Supplement: Supplementary file 3 — Reporting Summary [file 41467_2023_42814_MOESM3_ESM.pdf]

nature portfolio

Corresponding author(s): Zhen Wang

Last updated by author(s): Sep 13, 2023

Reporting Summary

Nature Portfolio wishes to improve the reproducibility of the work that we publish. This form provides structure for consistency and transparency in reporting. For further information on Nature Portfolio policies, see our Editorial Policies and the Editorial Policy Checklist.

Statistics

For all statistical analyses, confirm that the following items are present in the figure legend, table legend, main text, or Methods section.

n/a	Confirmed	
☐	☒	The exact sample size (n) for each experimental group/condition, given as a discrete number and unit of measurement
☐	☒	A statement on whether measurements were taken from distinct samples or whether the same sample was measured repeatedly
☐	☒	The statistical test(s) used AND whether they are one- or two-sided<br>*Only common tests should be described solely by name; describe more complex techniques in the Methods section.*
☐	☒	A description of all covariates tested
☐	☒	A description of any assumptions or corrections, such as tests of normality and adjustment for multiple comparisons
☐	☒	A full description of the statistical parameters including central tendency (e.g. means) or other basic estimates (e.g. regression coefficient) AND variation (e.g. standard deviation) or associated estimates of uncertainty (e.g. confidence intervals)
☐	☒	For null hypothesis testing, the test statistic (e.g. F, t, r) with confidence intervals, effect sizes, degrees of freedom and P value noted<br>*Give P values as exact values whenever suitable.*
☒	☐	For Bayesian analysis, information on the choice of priors and Markov chain Monte Carlo settings
☒	☐	For hierarchical and complex designs, identification of the appropriate level for tests and full reporting of outcomes
☐	☒	Estimates of effect sizes (e.g. Cohen's d, Pearson's r), indicating how they were calculated

Our web collection on statistics for biologists contains articles on many of the points above.

Software and code

Policy information about availability of computer code

Data collection	For fMRI, Brain scanning was performed using the 3.0 T SIEMENS MAGNETOM Prisma.<br>For rats, all behavioral tests were conducted during the animals' light cycle, videotaped, and analyzed with video tracking software (Ethovision, Noldus, Netherlands). All images were obtained using FLUOVIEW FV1200 confocal microscopes (Olympus) and Olympus VS200. Optical-fiber-based Ca2+ recording was performed using a Multi-Channel Fiber Photometry Device (inper, Hangzhou, China). Neurons were visualized with an IR camera on an Olympus U-TLUIR, and whole-cell-patch clamp recordings (multiclamp 700B amplifier) were made.<br>No custom algorithms or software was used for data collection.
Data analysis	Resting-state fMRI data were processed using Data Processing & Analysis for Brain Imaging (DPABI_V3.0 software, http://www.restfmri.net) and Statistical Parametric Mapping Software (SPM12, http://www.l.ion.ucl.ac.uk/spm) in MATLAB 2019a. we defined two functional regions of interest (ROIs) in our analysis: the left and right red nucleus (RN), and we used WFU PickAtlas software to generate the ROIs of the RN.<br>For rats, digitalized images were analyzed using Fuji (NIMH, Bethesda MD, USA).Enrichment between gene lists was analyzed using DAVID (2021 Update). Heatmap and volcano plot analyses of expression levels were analyzed using R4.2.0.All fiber photometry data and behavior videos were aligned offline thorough event markers using Inper Data Process (inper, Hangzhou, China) and analyzed by metlab2022a.Clinical data were analyzed using SPSS statistics 26. All animal data were analyzed for statistical significance using GraphPad Prism software.<br>No custom algorithms, code or software was used for data analysis.

For manuscripts utilizing custom algorithms or software that are central to the research but not yet described in published literature, software must be made available to editors and reviewers. We strongly encourage code deposition in a community repository (e.g. GitHub). See the Nature Portfolio guidelines for submitting code & software for further information.

Data

Policy information about availability of data

All manuscripts must include a data availability statement. This statement should provide the following information, where applicable:

- Accession codes, unique identifiers, or web links for publicly available datasets
- A description of any restrictions on data availability
- For clinical datasets or third party data, please ensure that the statement adheres to our policy

Source data are provided with this paper. All the data associated with this study are present in the paper or the Supplementary Materials. The RNA-seq raw data in this study have been deposited in Big Sub under the accession code GSE171321.

Human research participants

Policy information about studies involving human research participants and Sex and Gender in Research.

Reporting on sex and gender	In the study, 47 stressed individuals (mean age 40.79 ± 6.144 years) and 41 controls (mean age 41.05 ± 6.499) completed the survey. Gender, age, marriage status and education years had no difference between these two groups.
Population characteristics	Sex, age, marriage status and education years were not different between these two groups (Table 1).
Recruitment	we recruited psychiatrists who worked on the front line during the COVID-19 pandemic in Wuhan between February 21 and March 31, 2020. Psychiatrists who worked in non-COVID-19 second-line care were recruited as controls at the baseline interview. This clinical study was approved by the Ethics Committee of Shanghai Mental Health Center (2020-10). Written informed consent was provided by all participants prior to their enrollment. All human participants in our research underwent COVID-19 PCR/antigen testing, and the results were negative.
Ethics oversight	This clinical study was approved by the Ethics Committee of Shanghai Mental Health Center (2020-10).

Note that full information on the approval of the study protocol must also be provided in the manuscript.

Field-specific reporting

Please select the one below that is the best fit for your research. If you are not sure, read the appropriate sections before making your selection.

☒ Life sciences ☐ Behavioural & social sciences ☐ Ecological, evolutionary & environmental sciences

For a reference copy of the document with all sections, see nature.com/documents/nr-reporting-summary-flat.pdf

Life sciences study design

All studies must disclose on these points even when the disclosure is negative.

Sample size	We calculated the sample size using G*Power software. We set the α error probability to 0.05, power to 0.8, and number of groups to 3. We determined that a total sample size of 24 would be necessary. All tests were calculated using power analysis, and the power (1-β error probability) was greater than 0.8.
Data exclusions	No data were excluded.
Replication	All tests were repeated at least 3 times and all attempts at replication were successful.
Randomization	Allocation was random for animal research. For human research, stress group: psychiatrists who working in frontline during COVID-19 pandemic in Wuhan between February 21 and March 31, 2020; control group: the psychiatrists who working in non-COVID-19 second-line.
Blinding	The investigators were blinded to group allocation during data collection and analysis.

Reporting for specific materials, systems and methods

We require information from authors about some types of materials, experimental systems and methods used in many studies. Here, indicate whether each material, system or method listed is relevant to your study. If you are not sure if a list item applies to your research, read the appropriate section before selecting a response.

Materials & experimental systems

n/a	Involved in the study
☐	☒ Antibodies
☒	☐ Eukaryotic cell lines
☒	☐ Palaeontology and archaeology
☐	☒ Animals and other organisms
☒	☐ Clinical data
☒	☐ Dual use research of concern

Methods

n/a	Involved in the study
☒	☐ ChIP-seq
☐	☒ Flow cytometry
☐	☒ MRI-based neuroimaging

Antibodies

Antibodies used	CCL5, Catalogue #710001, Thermo Fisher; eIF4E Catalogue #66655 Proteintech; IRF-1 Catalogue #11335 Proteintech; vGlu1 Catalogue #48-2400 Invitrogen; GAD Catalogue #PA5-21397 Invitrogen; Alexa fluor 647-anti-mouse A-21245 Invitrogen; Alexa fluor 488-anti-rabbit 1796375 Invitrogen; Alexa fluor 555-anti-rabbit Invitrogen.
Validation	CCL5(This antibody is predicted to react with rat and human based on sequence homology, https://www.thermofisher.cn/order/genome-database/dataSheetPdf?producttype=antibody&productsubtype=antibody_primary&productId=710001&version=337), eIF4E(KD/KO validated, Reactivity: Human, Mouse, Rat; Applications: FC, IF, IHC, WB, ELISA. Publications: 2. https://www.ptgcn.com/products/EIF4E-Antibody-66655-1-Ig.htm),<br>IRF-1 (KD/KO validated, Reactivity: Human, Mouse, Rat; Applications: FC, IF, IHC, WB, ELISA. Publications:25. https://www.ptgcn.com/products/IRF1-Antibody-11335-1-AP.htm)<br>vGlu1 (This Antibody was verified by Cell treatment to ensure that the antibody binds to the antigen stated. Reactivity: Human, Mouse, Rat; Applications: IF, IHC, WB. Publications:23. https://www.thermofisher.cn/cn/zh/antibody/product/VGLUT1-Antibody-Polyclonal/48-2400)<br>GAD (Reactivity: Human, Mouse, Rat; Applications: IF, IHC, WB. Publications:4. https://www.thermofisher.cn/cn/zh/antibody/product/GAD67-Antibody-Polyclonal/PA5-21397)

Animals and other research organisms

Policy information about studies involving animals; ARRIVE guidelines recommended for reporting animal research, and Sex and Gender in Research

Laboratory animals	SD rats (Jackson Labs) at PND 64-90 were used in our experiments.
Wild animals	No wild animals were used in the study.
Reporting on sex	Both male and female rats were used in our study.
Field-collected samples	The study did not involve samples collected from the field.
Ethics oversight	All procedures involving rats were approved by the Institutional Animal Care and Use Committee at Shanghai Jiao Tong University and in accordance with the National Institutes of Health guidelines.<br>This clinical study was approved by the Ethics Committee of Shanghai Mental Health Center (2020-10).

Note that full information on the approval of the study protocol must also be provided in the manuscript.

Flow Cytometry

Plots

Confirm that:

☒ The axis labels state the marker and fluorochrome used (e.g. CD4-FITC).

☒ The axis scales are clearly visible. Include numbers along axes only for bottom left plot of group (a 'group' is an analysis of identical markers).

☒ All plots are contour plots with outliers or pseudocolor plots.

☒ A numerical value for number of cells or percentage (with statistics) is provided.

Methodology

Sample preparation	1. Wash the sample with cold D-PBS, cut it into small pieces of about 0.5cm after cleaning, put it into tube C, add 1950 ul of mixed enzyme 1 and 30 ul of mixed enzyme 2 into tube C, cover tube C tightly, Transfer to a tissue dissociation apparatus.<br>2. Run the program "37C_ABDK_01". After the dissociation, filter with a 70um filter, add 10ml of cold D-PBS to the filtered tube C, wash down the remaining samples in the tube C, and filter. Centrifuge to collect the precipitate.<br>3. Select the ratio according to the weight of the sample. According to the ratio, first add D-PBS to resuspend the cell pellet, then add Debris Removal Solution to mix with the sample, transfer to a 5ml or 15ml centrifuge tube, and spread a layer of D-

	PBS on the mixture. PBS. 3000rcf, 4°C, 10min centrifugation, full up full down.<br>4. After centrifugation. Remove the upper two layers, add 10ml D-PBS to the tube, centrifuge at 1000rcf, 4°C, 10min, wash off the residual liquid, pour off the supernatant, and collect the precipitate.<br>5. Wash the cells twice with cold PBS buffer, and then make a suspension of 1X 10 7 cells/ml with 1X PBS buffer.
Instrument	flow cytometry using Celesta (BD)
Software	FlowJo_V10
Cell population abundance	Wash the cells twice with cold PBS buffer, and then make a suspension of 1X 107 cells/ml with 1X PBS buffer.
Gating strategy	Forward and side scatter density plots for identifying your cell population of interest and excluding debris. FSC intensity is proportional to the diameter of the cell, and is primarily due to light diffraction around the cell. Forward scatter is detected by a photodiode, which converts the light into an electrical signal. The intensity of the produced voltage is proportional to the diameter of the interrogated cell. Side scatter (SSC) measurement provides information about the internal complexity of a cell.

☐ Tick this box to confirm that a figure exemplifying the gating strategy is provided in the Supplementary Information.

Magnetic resonance imaging

Experimental design

Design type	Resting state
Design specifications	Eighty-three participants were recruited, including forty-four stressed psychiatrists and thirty-nine demographically matched controls. Brain scanning were performed using 3.0T SIEMENS MAGNETOM Prisma at Renji Hospital.
Behavioral performance measures	n/a

Acquisition

Imaging type(s)	Resting-state functional images and Structural MRI images
Field strength	3.0T
Sequence & imaging parameters	Resting-state functional images were acquired using acquisition time 452 s, repetition time 2000 ms, echo time 30 ms, flip angle 90°, voxel size 3.3*3.6*2.4 mm, field of view 230*230 mm2, number of slices 70 and 220 time points. Structural MRI images were also acquired using acquisition time 221 s, repetition time 1800 ms, echo time 2.28 ms, flip angle 8°, voxel size 1*1*1 mm, field of view 256*256 mm2, number of slices 160.
Area of acquisition	The first 10 volumes were removed, leaving 210 volumes for each participant. Nuisance covariates, including Friston 24-parameter, white matter, and cerebrospinal fluid signals, were regressed out. The structural images were checked one by one and manually re-oriented to align the anterior commissure to the origin, and then co-registered to the mean functional images.

Diffusion MRI ☐ Used ☒ Not used

Preprocessing

Preprocessing software	Resting-state fMRI data were processed using Data Processing & Analysis for Brain Imaging (DPABI_V3.0 software, http://www.restfmri.net) and Statistical Parametric Mapping Software (SPM12, http://www.l.ion.ucl.ac.uk/spm) in MATLAB 2019a.
Normalization	The correlation r-maps were converted into Z-maps by Fisher's r-to-z transformation. The full factorial model was used to examine the rsFC differences in the seven seeds among the three groups by SPM12 in the general linear model, with mean FD as covariates.
Normalization template	The structural images were checked one by one and manually re-oriented to align the anterior commissure to the origin, and then co-registered to the mean functional images. They were then segmented as grey matter, white matter and cerebrospinal fluid by the Diffeomorphic Anatomical Registration Through Exponentiated Lie Algebra (DARTEL) algorithm (Ashburner, 2007). Then, the functional images were normalized with DARTEL into the standard Montreal Neurological Institute (MNI) Space in 3mmx 3mm x 3mm voxel size by applying the parameters obtained during segmentation. Smoothing by a Gaussian kernel with 4mm full-width at half-maximum value (FWHM) was then carried out. Finally, temporal band-pass filtering (0.01-0.1 Hz) was performed. In order to exclude the potential effect of the head motion, the mean frame-wise displacement (FD), a relative movement parameter, was calculated as a covariate in the subsequent analysis.
Noise and artifact removal	We excluded participants whose maximum head motions during the resting-state fMRI scan were greater than 2mm or had a rotation greater than 2° based on absolute movement in the preprocessing analysis.
Volume censoring	We used the WFU PickAtlas software to generate the functional regions-of-interest (ROIs) of bilateral red nucleus (RN). Then we used the bilateral RN as ROIs to conduct voxel-wise rsFC analysis between each seed and the whole brain voxels by DPABI.

Statistical modeling & inference

Model type and settings	model univariate; fixed effects
Effect(s) tested	The rsFC differences among the EPR and controls were examined. The clusters were considered as exhibiting significant group differences if they reached a voxel-level threshold of $P < 0.001$ with cluster-level P (FDR corrected) < 0.05 and had a cluster size of > 30 voxels.

Specify type of analysis: ☐ Whole brain ☒ ROI-based ☐ Both

Anatomical location(s)	We used the WFU PickAtlas software to generate the functional regions-of-interest (ROIs) of bilateral red nucleus (RN). Then we used the bilateral RN as ROIs to conduct voxel-wise rsFC analysis between each seed and the whole brain voxels by DPABI. The mean time series of the seeds were calculated and correlated with the time series of all other whole brain voxels.
Statistic type for inference (See Eklund et al. 2016)	The correlation r-maps were then converted into Z-maps by Fisher's r-to-z transformation. The full factorial model was used to examine the rsFC differences in the seven seeds among the three groups by SPM12 in the general linear model, with mean FD as covariates.
Correction	FDR

Models & analysis

n/a	Involved in the study
☐	☒ Functional and/or effective connectivity
☒	☐ Graph analysis
☒	☐ Multivariate modeling or predictive analysis

Functional and/or effective connectivity	Pearson correlation
